# *ESR1* overexpression is a biomarker of relapse and worse prognosis in stage I endometrioid endometrial carcinoma

**DOI:** 10.1590/1414-431X2025e14494

**Published:** 2025-05-09

**Authors:** C.B.P. Chaves, P. Nicolau-Neto, T.A. Simão, P.T. de Souza-Santos, A. Bergmann, L. Brewer, F.C.B. Moreira, B.S.B. Reis, M.A.M. Moreira, L.F.R. Pinto

**Affiliations:** 1 Seção de Ginecologia Oncológica, Divisão de Pesquisa Clínica e Inovação Tecnológica, Instituto Nacional de Câncer - INCA, Rio de Janeiro, RJ, Brasil; 2 Programa de Carcinogênese Molecular, Instituto Nacional de Câncer - INCA, Rio de Janeiro, RJ, Brasil; 3 Departamento de Bioquímica, Instituto de Biologia Roberto Alcântara Gomes, Universidade do Estado do Rio de Janeiro, Rio de Janeiro, RJ, Brasil; 4 Biologia Molecular - Beneficência Portuguesa de São Paulo, São Paulo, SP, Brasil; 5 Programa de Epidemiologia Clínica, Instituto Nacional de Câncer - INCA, Rio de Janeiro, RJ, Brasil; 6 Divisão de Patologia, Instituto Nacional de Câncer - INCA, Rio de Janeiro, RJ, Brasil; 7 Divisão de Genética, Instituto Nacional de Câncer - INCA, Rio de Janeiro, RJ, Brasil

**Keywords:** Endometrial cancer, Early stage, Tumor relapse, Prognosis, ESR1

## Abstract

Endometrial cancer (EC) is the most common pelvic gynecologic malignancy in developed countries, and its incidence is also increasing in developing countries. Endometrioid endometrial carcinoma (EEC) is the most frequent subtype. EEC is often associated with favorable clinicopathological features and a good prognosis, especially when diagnosed in stage I. Although some patients have no signs to predict locally advanced or metastatic disease, they may present tumor relapse in the future. There is no biomarker capable of predicting the relapse of stage I EEC. The present study applied a transcriptome analysis to identify differentially expressed genes in stage I EEC, comparing relapsed with non-relapsed tumors. The estrogen receptor 1 gene (*ESR1*) was overexpressed in EEC stage I samples from patients who developed relapse by 4.3-fold compared to non-relapsed tumors. Subsequently, an independent set of 64 stage I EEC samples was used to validate *ESR1* gene overexpression in relapsed tumors and assess estrogen receptor alpha (ERα) protein levels. *ESR1* was confirmed to be overexpressed in samples from relapsed tumors, and its expression level was an independent prognostic variable for disease-free (hazard ratio=7.25) and overall survival (hazard ratio=5.15). In contrast, Erα did not show different values between relapsed and non-relapsed tumors. We concluded that *ESR1* overexpression is a biomarker for poor prognosis in stage I EEC.

## Introduction

Endometrial cancer (EC) is the sixth most common cancer among women worldwide. EC incidence is higher in developed countries and is increasing in developing countries (1,2). Endometrial cancer is classified into two subtypes with different clinicopathological features and prognoses. Type I, or endometrioid endometrial carcinoma (EEC), is the most frequent subtype and often shows a good prognosis. In contrast, type II tumors, or non-endometrioid carcinomas, represent the minority of endometrial cancers and are related to aggressive spread and poor outcomes (3,4). There are also differences in genetic alterations between EC subtypes, although these changes alone do not explain differences in tumor behavior and outcomes (5,6).

Favorable clinicopathological features in stage I EEC tumors, such as low-grade and superficial myometrial invasion, usually confer a good prognosis. However, some patients (ranging from 6 to 22%) may present an unexpected recurrence with limited responsiveness to systemic therapy (7-9). Unfortunately, no feature or potential biomarker can predict the recurrence of stage I EECs. Therefore, this study aimed to identify a biomarker that can predict the recurrence of stage I EEC.

In the present study, we analyzed the transcriptome of stage I EECs with and without recurrence, aiming to find genes differentially expressed and associated with relapse prediction.

## Material and Methods

### EEC samples for transcriptome analysis and validation

Patients included in this study participated in a retrospective cohort of stage I EEC, with samples stored in the National Tumor Bank and at the Pathology Division of the Brazilian National Cancer Institute (INCA). They were treated at the Gynecological Oncology Department between 2000 and 2011, and clinicopathological features were reviewed in medical records. Patients underwent total abdominal hysterectomy and bilateral salpingo-oophorectomy. In addition, pelvic lymphadenectomy and removal of enlarged para-aortic lymph nodes were performed according to established criteria (10). None of the patients had received radiation, chemotherapy, or hormonal treatment before surgery. Patients were followed for 60 months to check for tumor relapse. Tumor relapse was characterized as local/loco-regional recurrence or distant metastasis after surgical treatment.

Samples were allocated into two groups: investigation set and validation set. For the investigation set, ten snap-frozen (at -80°C until processing) samples were collected, with five from patients who presented relapse and five from patients who did not present relapse. For the validation set, 64 formalin-fixed paraffin-embedded (FFPE) samples were collected, with 22 from patients with relapse and 42 from patients who did not have relapse. All samples were analyzed by a pathologist who confirmed the presence of at least 80% tumor cells. The institution's Ethics Committee approved this study (approval number 13338719.3.0000.5274), and all patients signed the Informed Consent for study participation. EEC staging was based on the 2009 International Federation of Gynecology and Obstetrics (FIGO) criteria (11).

### RNA extraction and quality control

In the investigation set, RNA was extracted from frozen tissue using the RNeasy Mini Kit (Qiagen, Germany), according to the manufacturer's instructions. RNA purity was determined using an RNA Nano Chip in the Agilent 2100 Bioanalyzer (Agilent Technology, USA), and only samples with an RNA integrity number (RIN) ≥8 were included for transcriptomic analysis.

RNA was extracted from FFPE samples for the validation set, with ten 10-μm sections per sample, using the RNeasy FFPE Kit (Qiagen) following the manufacturer's protocol. The RNA samples were quantified in the Nanodrop system (Thermo, USA). To ensure quality control of the RNA extracted from FFPE samples, successful *GAPDH* amplification by qPCR was mandatory for subsequent analyses. Therefore, all samples that showed *GAPDH* amplification were included in this study.

### Gene expression profiling

Transcriptome analyses were performed with the samples of the investigation set using microarray as previously described (12). Biotinylated cDNA of each sample was applied to each Human Exon 1.0 ST array (Thermo). The raw data was normalized in the Expression Console software (Thermo) using the robust multi-array average (RMA) (13). Subsequent analyses of gene expression data were carried out in the Transcriptome Analysis Console software (Thermo), using the *limma* eBays method. The criteria to determine differentially expressed genes (DEG) were expression fold-change ≥±2.0 and P-value <0.05. Microarray data are available at Gene Expression Omnibus (GSE225956) database. Computing language R was used for the Bayesian Hierarchical Clustering of described DEG.

### Enrichment analysis of differentially expressed genes

Post-processing analysis of DEGs was performed on the WEB-based gene set analysis toolkit (14), and the potential main transcriptional factors were assessed. For this purpose, a hypergeometric test was used for enrichment evaluation analysis with a P-value <0.001 as the statistical parameter, performing the Benjamini & Hochberg method for multiple test adjustments.

### Gene expression validation by qPCR

Quantitative RT-PCR (RT-qPCR) was performed to validate DEG data. cDNA was synthesized from 3.0 μg RNA of each sample by SuperScript II Reverse Transcriptase (Invitrogen, USA). qPCR was performed in 15.0 μL reaction volume with Rotor-Gene SYBR Green PCR kit (Qiagen) on an RG 6000 thermal cycler (Qiagen). Samples were analyzed in triplicate. mRNA relative quantification was investigated by the ΔCt method using *GAPDH* as a housekeeping gene (15). The primers used were: ESR1-F 5′-GCTCCTTTCTCCTGCCCATT-3′; ESR1-R 5′-TCCTTCCTAGTTTTCTTCTTCTTGA-3′ (amplicon size 84 bp); GAPDH-F 5′-CAACAGCCTCAAGATCATCAGCAA-3′; and GAPDH-R 5′-AGTGATGGCATGGACTGTGGTCAT-3′ (amplicon size 124 bp).

### Immunohistochemistry

Estrogen receptor alpha (ERα) expression was assessed in the validation set of samples. Deparaffinized and rehydrated sections (3.0 µm) of EEC paraffin-embedded tissues were mounted on coated slides. Antigen retrieval was accomplished by immersing slides in EDTA buffer, pH=9.0, for 30 min at 98°C in a water bath. Immunohistochemistry staining was done at 4°C overnight, with a monoclonal antibody against estrogen receptor (ER), rabbit monoclonal clone SP1, and SPRING Bioscience M3014. The detection was performed by Novolink™ Max Polymer Detection System (Leica, Germany) following the manufacturer's instructions. Harris' hematoxylin was used to stain the slides (Merck, Germany). ER-positive breast cancer samples were used as a positive control, and the absence of primary antibody was the negative control. Two independent pathologists examined all cases. The ERα was classified as 0 (negative) when the immunohistochemical nuclei staining was <1%; 1+ when nuclei stained positive was >1 and ≤20%; 2+ for cases with nuclei staining >20 and ≤50%; and 3+ when over 50% of nuclei of the tumor cells were stained.

### Statistical analyses

Association analyses between tumor relapse and clinicopathological characteristics (age, body mass index (BMI), comorbidities (hypertension and diabetes), adjuvant treatment, myometrial invasion, tumor grade, and lymphovascular invasion) were performed using Pearson chi-squared test. Gene expression analysis using RT-qPCR data was performed in GraphPad Prism 5.0 software. As the expression values in the samples were not normally distributed, the Mann-Whitney test was applied, and results were considered significant when P<0.05. Gene expression sensitivity and specificity to distinguish EEC cases that presented recurrence from those that did not were measured by the receiver operating characteristic (ROC) curve.

Survival analyses were performed with a 5-year follow-up after surgery, using the Kaplan-Meier method and log-rank test. Patient death was the event in the overall survival (OS) analysis, and tumor relapse (local, regional, or distant) was considered in the disease-free survival (DFS) analysis. In cases without event, the last registration date in the medical record was used, and the follow-up was censored. Variables with a P-value lower than 0.2 were selected for multivariate analyses. Cox regression model using the stepwise forward method was used to estimate the independent factors associated with outcome. The variables retained in the final model were used to adjust the risk of recurrence or death associated with *ESR1* expression. For these analyses, the statistically significant P-values were <0.05. Survival analyses were performed in R using the survival package.

## Results

### Clinicopathological data

Among patients in the investigation set (n=10), four women were older than 65 years and nine were obese or overweight. Most of them had hypertension (n=8), including five with concomitant diabetes. None of them were under hormone replacement therapy (HRT), half used birth control pills (BCP), and nine had confirmed previous pregnancies. Five cases were diagnosed with stage IA (two of which presented relapse) and another five cases were stage IB (three of which presented recurrence). One tumor had lymphovascular invasion, and this patient relapsed. Six tumors were low-grade and four were high-grade. Sixty percent of the tumors were larger than 5.0 cm. There were two deaths; one patient was diagnosed with stage IA and the other with stage IB EEC. In the investigation set, relapse occurrence was only associated with tumor size (P=0.047) (Supplementary Table S1).

In the validation set (n=64), there was no difference in the median age of patients with or without relapse (63.5 and 64 years, respectively, P=0.817) or in any other clinicopathological characteristic, except diabetes, which was more common among patients who did not have relapse (P=0.023) (Table 1).

**Table 1:** Clinicopathological characteristics of stage I endometrioid endometrial carcinoma (EEC) patients included in the validation set, comparing non-relapsed (n=42) and relapsed cases (n=22).

Variable	Non-relapsed (n^a^, %)	Relapsed (n^a^, %)	P-value^a^
Age (years)			
≤65	23 (54.8)	14 (63.6)	0.598
>65	19 (45.2)	8 (36.4)	
BMI			
Adequate	8 (19.0)	2 (9.1)	0.471
Overweight/obesity	32 (76.2)	18 (81.8)	
No information	2 (4.8)	2 (9.1)	
Hypertension			
No	14 (33.3)	7 (31.8)	
Yes	28 (66.7)	15 (62.2)	1.000
No information	0	0	
Diabetes			
No	29 (69.0)	21 (95.5)	
Yes	13 (31.0)	1 (4.5)	0.023
No information	0	0	
Pre-menopause			
No	31 (73.8)	17 (77.3)	
Yes	5 (11.9)	3 (13.6)	
No information	6 (14.3)	2 (9.1)	1.000
Pregnancy			
No	4 (9.5)	4 (18.9)	0.423
Yes	38 (90.5)	17 (77.3)	
No information	0	1 (2.8)	
HRT			
No	21 (50.0)	11 (50.0)	
Yes	6 (14.3)	0	0.154
No information	15 (35.7)	11 (50.0)	
BCP			
No	16 (38.1)	5 (22.7)	0.494
Yes	12 (28.6)	7 (31.8)	
No information	14 (33.3)	10 (45.5)	
Stage			
IA	22 (52.4)	9 (40.9)	0.438
IB	20 (47.6)	13 (59.1)	
Lymphovascular invasion			
No	34 (87.2)	17 (77.3)	0.473
Yes	5 (12.8)	5 (22.7)	
Grade			
1+2	38 (90.5)	16 (72.7)	
3	4 (9.5)	6 (27.3)	0.080
Tumor size (cm)			
≤5.0	27 (64.3)	9 (40.9)	0.077
>5.0	11 (26.2)	10 (45.5)	
No information	4 (9.5)	3 (13.6)	

^a^Calculated with known values; chi-squared test. BMI: body mass index; HRT: hormone replacement therapy; BCP: birth control pill.

The frequency of clinicopathological features between investigation and validation sets of samples was also compared, and no significant differences were observed, including the frequency of relapse (Supplementary Table S2).

### Gene expression profile associated with recurrence in stage I EEC

**Figure 1:**
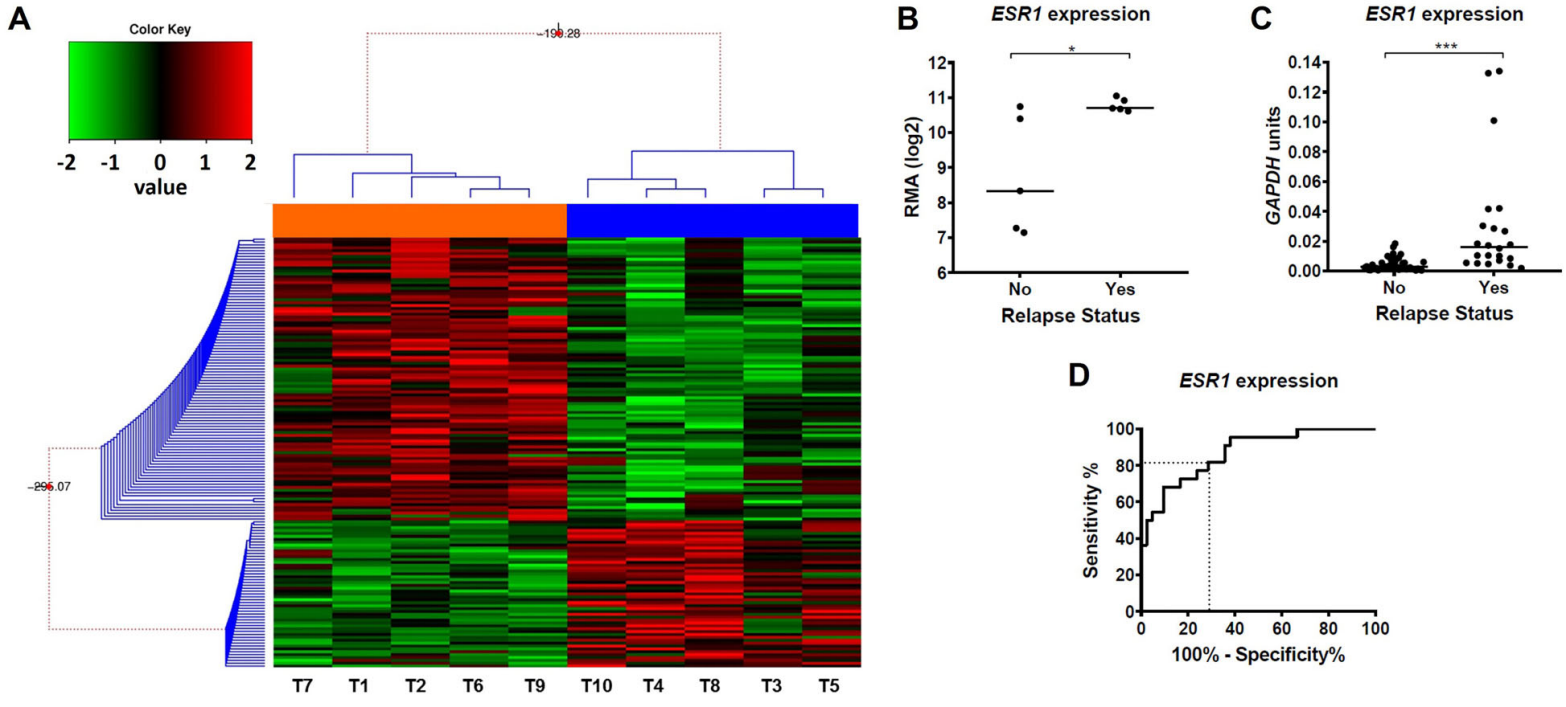
Transcriptomic analysis of endometrioid endometrial carcinoma (EEC) showing *ESR1* overexpression as a relapse biomarker. **A**, Expression data of the 149 differentially expressed genes (DEGs) were used for Bayesian hierarchical clustering. Samples were clustered into two groups: ECC patients with relapsed (orange bars) and non-relapsed tumors (blue bars), without misclassification. Each column represents an individual sample and each line represents a gene. The red and green colors represent increased and decreased gene expression, respectively. **B** and **C**, *ESR1* expression was assessed in the investigation (**B**) and validation (**C**) samples, showing overexpression in stage I EEC samples from patients with relapse. *ESR1* expression was 4.3-fold higher in the relapsed stage I EEC group compared to the non-relapsed group in the investigation set of samples (P<0.030) and in the validation set of samples (P=0.001). **D**, ROC curve shows that *ESR1* expression can discriminate stage I EEC samples from patients with or without relapse with an accuracy of 0.87%, a sensitivity of 65.9%, and a specificity of 81.4%.

The hierarchical clustering of EEC samples using the DEG data shows two distinct groups: a cluster composed of tumors from patients with and without relapse (Figure 1A). Transcriptome analysis pointed out 149 DEGs between relapsed and non-relapsed EEC. Among them, 98 were overexpressed and 51 were underexpressed in relapsed EEC samples (Supplementary Table S3). *ESR1* (expression fold-change=4.29; P=0.03) and *PGR* (expression fold-change=37.7; P=0.002), which encode the estrogen receptor alpha (ERα) and progesterone receptor, respectively, were overexpressed in relapsed stage I EEC.

Aiming to identify possible regulators of gene expression reprogramming in the group of relapsed stage I EEC samples, prediction analysis of transcription factors (TF) that could regulate DEG transcriptional profile was performed, and 27 TF were identified (Table 2). Among these, *ESR1* was selected for the following analyses.

**Table 2:** Transcriptional factor network analysis for potential regulators of differentially expressed genes (DEGs) in relapsed-stage I endometrioid endometrial carcinoma (EEC).

Transcriptional factor	Gene symbol	Database members	Observed	Expected	Adjusted (P-value)
FOXO4	*FOXO4*	2037	32	7.04	1.89e-10
E12	*E12*	2450	34	8.46	4.90e-10
LEF1	*LEF1*	1939	30	6.70	5.60e-10
AP1	*AP1*	1104	19	3.81	9.86e-07
MAS	*MAZ*	2250	24	7.77	5.45e-05
TTANTCA_UNKNOWN	-	937	15	3.24	5.45e-05
SP1	*SP1*	2891	27	9.99	0.0001
MEF2	*MEF2*	691	12	2.39	0.0002
FOX	*FOX*	208	7	0.72	0.0003
PAX4	*PAX4*	1278	16	4.42	0.0003
AACTTT_UNKNOWN	-	1859	20	6.42	0.0003
FAC1	*BPTF*	219	7	0.76	0.0004
NFAT	*NFAT*	1871	19	6.46	0.0005
AREB6	*ZEB1*	780	12	2.69	0.0005
CHX10	*VSX2*	797	12	2.75	0.0005
SREBP1	*SREBP1*	245	7	0.85	0.0005
TCF4	*TCF4*	240	7	0.83	0.0005
FREAC2	*FOXF2*	907	13	3.13	0.0005
PITX2	*PITX2*	582	10	2.01	0.0006
P53	*TP53*	253	7	0.87	0.0006
TATA	*TBP*	1276	15	4.41	0.0006
NF1	*NF1*	709	11	2.45	0.0006
CEBP	*CEBP*	265	7	0.92	0.0006
BACH1	*BACH1*	258	7	0.89	0.0006
**ER**	***ESR1***	**264**	**7**	**0.91**	**0.0006**
HNF3	*FOXA*	733	11	2.53	0.0007
HOXA9	*HOXA9*	117	5	0.40	0.0007

*ESR1* was selected for the further analyses and is shown in bold type.

### *ESR1* expression could predict recurrence in stage I EEC

*ESR1* gene was overexpressed in stage I EEC with recurrence (4.3-fold, P=0.03) compared to non-recurrence cases in the investigation set of samples (Figure 1B). Further, *ESR1* expression was also confirmed by RT-qPCR in the validation set of samples, showing a median 4.3-fold higher expression in tumors that presented recurrence compared to non-recurrence tumors (P<0.001) (Figure 1C). Finally, the sensitivity and specificity of *ESR1* to predict recurrence were assessed by the gene expression values in a ROC curve. The *ESR1* expression cutoff of 3.91×10^-3^ relative to *GAPDH* showed 81.4% specificity and 65.9% sensitivity, with an 87% accuracy in distinguishing tumors by recurrence status (Figure 1D).

Association analyses between *ESR1* expression and clinicopathological features showed higher *ESR1* expression in EEC patients with no diabetes history (Supplementary Table S4).

### ER**α** protein expression in EEC

The evaluation of ERα protein expression was also assessed in the 64 samples, with 58 (90.62%) considered positive and 6 (9.38%) considered negative. Among positive samples, 17 (26.56%) were 1+, 15 (23.44%) were 2+, and 26 (40.62%) were 3+. In the group of 42 non-relapsed stage I EEC, three (7.14%) were negative, 10 (23.83%) were considered 1+, 9 were classified as 2+ (21.42%), and 20 were 3+ (47.61%). In the sample of relapsed tumors (n=22), 3 (13.64%) were considered negative, 7 (31.82%) were classified as 1+, 6 (27.27%) were 2+, and 6 (27.27%) were 3+ (Supplementary Figure S1).

The relationship between ERα protein expression frequencies, clinicopathological characteristics, and prognosis was investigated. There was no association between ERα protein expression and the clinical features of EEC patients. Regarding the anatomopathological characteristics, patients with G1 and G2 tumors had a higher chance of being ERα positive than G3 tumors (OR=7.28; P=0.044; 95%CI=1.40-34.48). Recurrence was not statistically associated with ERα protein expression (P=0.40). Finally, the association between *ESR1* mRNA and ERα protein expression was evaluated, and no statistically significant linear or non-linear correlation was observed (P=0.99).

To better understand potential mechanisms of *ESR1* gene expression regulation in stage I EEC, we conducted a correlation analysis, using the microarray data, between *ESR1* and the genes that encode transcription factors described above (Table 2). No significant correlation was observed in this analysis (Supplementary Table S5). However, it is worth highlighting the potential correlation between *ESR1* and *FOXA2* (r=0.62; P=0.06).

### *ESR1* overexpression was associated with a worse prognosis

**Figure 2:**
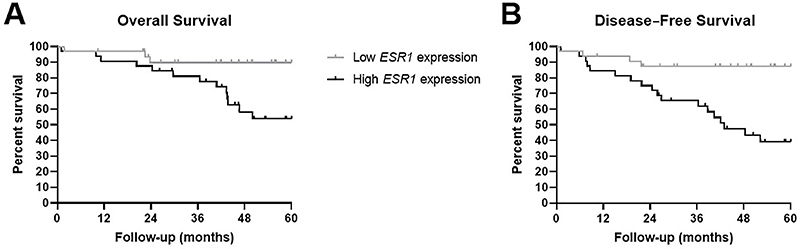
Kaplan-Meier curves showing *ESR1* gene overexpression associated with worse overall survival rates (**A**, P-value=0.02; HR=4.45) and disease-free survival rates (**B**, P-value=0.003; HR=5.48) in women with stage I endometrioid endometrial carcinoma (EEC). High and low *ESR1* expressions were calculated according to the median value. HR: hazard ratio.

We evaluated if the *ESR1* gene and protein expression might affect the outcome of stage I EEC patients. In the analysis with gene expression data, samples were classified according to the *ESR1* median expression value. Univariate analysis indicated a positive association between high expression of the *ESR1* gene and worse prognosis in DFS (P=0.001) and OS (P=0.013) rates, increasing the risk of relapse by 5.48-fold and the risk of death by 4.19-fold (Figure 2). In multivariate analysis, which included the variables age at diagnosis, myometrial invasion, and lymphovascular invasion, high *ESR1* expression was also reported as an independent prognostic factor for DFS (HR=7.25; P=0.002; 95%CI=1.99-26.34) and OS (HR=5.15; P=0.035; 95%CI=1.11-3.69) (Supplementary Table S6). Tumor grade was also an independent prognostic factor, but only for DFS (HR=3.31; P=0.020; 95%CI=1.20-9.09). ERα protein expression was not associated with patient outcomes either in DFS (P=0.35) or OS (P=0.74).

## Discussion

Although stage I EEC is related to good outcomes, some patients develop recurrence, resulting in poorer survival rates (16-18). This study identified the transcriptomic reprogramming associated with recurrence in stage I EEC. Integrative analyses highlighted *ESR1* overexpression in tumors with relapse, showing that this biomarker may discriminate tumor groups according to recurrence status with high accuracy. *ESR1* overexpression was an independent prognostic marker for DFS and OS. Therefore, *ESR1* expression evaluation in stage I EEC could be useful in the management of those patients and treatment selection.

The association between *ESR1* alteration and EEC prognosis has been investigated in other studies. Wik et al. (19) observed an association between ESR1 low expression and worse disease-specific survival rates in a sample set with all stages. However, they did not analyze stage I tumors separately or evaluate DFS and OS. Furthermore, *ESR1* mutations were related to EEC development in low-risk women, conferring worse prognosis than in *ESR1*-wild-type EEC (20). In addition, Rahman et al. (21) showed that *ESR1* gene amplification is an independent factor for poor OS. They suggested it may be an early event in EEC development. In addition, somatic alterations and splice variants in *ESR1* have been associated with a hormone-independent activity, enabling ligand-binding independent transcriptional activity that seems to be associated with hormone-independent growth of breast and endometrial carcinomas (22-25).

This study showed an apparent discrepancy between estrogen receptor mRNA and protein levels and their association with prognosis. This discrepancy has already been observed in ovarian cancer, a tumor with similar risk factors to those related to EEC. The *ESR1* expression was significantly associated with patient prognosis, whereas ERα protein expression did not provide prognostic information (26). Other overexpressed genes, such as *TCOF1* and *DLGAP5*, but not their coded proteins, were associated with worse prognosis in EEC (27,28). Similar data was observed in breast cancer, in which *PDCD1* gene expression, but not PD-1 protein, was associated with the prognosis of triple-negative breast cancer (29). This could be explained by the dynamic control of mRNA translation, mediated by microRNAs, long non-coding RNAs, RNA-binding proteins (30), and epigenetic control of RNA expression (31). In addition, the availability of intracellular resources for protein synthesis and function could impact this scenario (32). Some microRNAs that are overexpressed in EEC, such as mir-632, mir-15b, and mir-331-3p, have already been associated with ERα expression, and this could explain the lack of a correlation between *ESR1* gene expression and ERα protein levels in our study and their association with recurrence and prognosis (33).

Our data highlighted the potential gene expression correlation of *ESR1* and *FOXA2*, a member of the FOX (forkhead box) transcriptional factor group. Similar data was observed by Sahoo et al. (34) in which the authors presented a positive and significant correlation between protein levels of ERα and FOXA2 in normal human endometrium and grade 1-3 human endometrial carcinomas.

EC can be molecularly classified into four distinct prognostic groups (35). However, this molecular classification was made by analyzing all tumor stages and histological subtypes. In our study, we evaluated only stage I EEC, which makes using this molecular classification unfeasible. In 2021, updated evidence-based guidelines were published, comprising a well-known risk stratification categorization including the EC molecular classification (36). Those recent findings changed the clinical practice guidelines of EC as a whole. Nevertheless, there is still much to be revealed about early EEC and poor prognosis.

A preliminary small EEC sample set was assessed in the transcriptomic analysis, which could be considered a limitation of our study. Nevertheless, the expression profile of the selected gene was validated in an independent set of samples, and relevant and reliable results regarding the association between *ESR1* overexpression and stage I EEC prognosis were obtained from our data.

In conclusion, this exploratory study showed that *ESR1* was overexpressed in stage I EEC that developed recurrence and was also a potential independent prognostic biomarker for EEC.

## Supplementary Material

Click here to view [pdf].
