# Supplementary material for: ESR1 overexpression is a biomarker of relapse and worse prognosis in stage I endometrioid endometrial carcinoma
Source: Braz J Med Biol Res. 2025 May 9;58:e14494. doi: 10.1590/1414-431X2025e14494 (PMC12068764; doi:10.1590/1414-431X2025e14494)
Supplement: Supplementary file 1 [file 1414-431X-bjmbr-58-e14494-suppl.pdf]

Figure S1. Estrogen receptor alpha (ERα) protein expression in endometrioid endometrial carcinoma (EEC) samples. ERα expression classification according to the nuclei staining percentage in EEC samples. **A**, Negative staining (<1% positive cells); **B**, 1+ positive (>1 and ≤20% positive cells); **C**, 2+ positive (>20 and ≤50% positive cells); **D**, 3+ positive (>50% positive cells). Scale bar 50 μm; magnification of 20×.

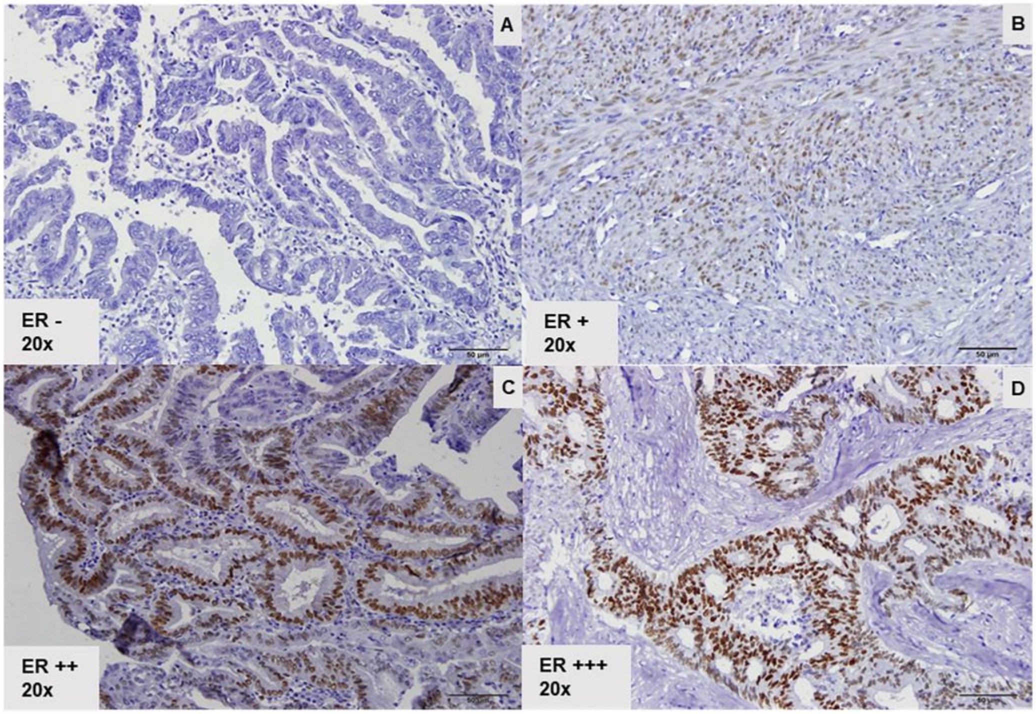

Table S1. Clinicopathological characteristics of stage I endometrioid endometrial carcinoma (EEC) patients included in the investigation set, comparing relapsed and non-relapsed cases.

Variable	Non-Relapsed (n[a], %)	Relapsed (n[a],%)	P-value[a]
Age (years)			
≤65	4 (80.0)	2 (40.0)	0.52
>65	1 (20.0)	3 (60.0)	
BMI			
Adequate	0 (0.0)	1 (0.0)	0.99
Overweight/obesity	5 (100)	4 (100)	
Hypertension			
No	1 (20.0)	1 (20.0)	1.00
Yes	4 (80.0)	4 (80.0)	
Diabetes			
No	3 (60.0)	2 (40.0)	0.99
Yes	2 (40.0)	3 (60.0)	
Pregnancy			
No	0 (0,0)	1 (20.0)	0.99
Yes	5 (100)	4 (80.0)	
HRT			
No	4 (80.0)	4 (80.0)	1.00
Yes	0 (0.0)	0 (0.0)	
No information	1 (20.0)	1 (20.0)	
BCP			
No	2 (40.0)	3 (60.0)	0.99
Yes	2 (40.0)	1 (20.0)	
No information	1 (20.0)	1 (20.0)	
Stage			
IA	3 (60.0)	2 (40.0)	0.99
IB	2 (40.0)	3 (60.0)	
Lymphovascular invasion			
No	5 (100)	4 (80.0)	0.99
Yes	0 (0.0)	1 (20.0)	
Tumor grade			
1+2	3 (60.0)	3 (60.0)	1.00
3	2 (40.0)	2 (40.0)	
Tumor size (cm)			
≤5.0	4 (80.0)	0 (0.0)	**0.047**
>5.0	1 (20.0)	5 (100)	

[a]Calculated with known values; chi-squared test. Bold type indicates statistically significant. BMI: body mass index; HRT: hormone replacement therapy; BCP: birth control pill.

Table S2. Clinicopathological characteristics of stage I endometrioid endometrial carcinoma (EEC) patients, comparing investigation and validation sets of samples.

Variable	Investigation (n[a],%)	Validation (n[a],%)	P-value[a]
Age (years)			
≤65	6 (60.0)	37 (57.8)	1.000
>65	4 (40.0)	27 (42.2)	
BMI			
Adequate	1 (10.0)	10 (15.6)	1.000
Overweight/obesity	9 (90.0)	50 (78.1)	
No information	0	4 (6.3)	
Hypertension			
No	2 (20.0)	21 (32.8)	
Yes	8 (80.0)	43 (67.2)	0.715
No information	0	0	
Diabetes			
No	5 (50.0)	50 (78.1)	
Yes	5 (50.0)	14 (21.9)	0.112
No information	0	0	
Pre-menopause			
No	7 (73.8)	48 (75.0)	
Yes	0 (11.9)	8 (12.5)	0.581
No information	3 (14.3)	8 (12.5)	
Pregnancy			
No	1 (10.0)	8 (12.5)	1.000
Yes	9 (90.0)	55 (85.9)	
No information	0	1 (1.6)	
HRT			
No	8 (80.0)	32 (50.0)	
Yes	0	6 (9.4)	0.571
No information	2 (20.0)	26 (40.6)	
BCP			
No	4 (40.0)	21 (32.8)	
Yes	4 (40.0)	19 (29.7)	1.000
No information	2 (20.0)	24 (37.5)	
Stage			
IA	5 (50.0)	31 (48.4)	1.000
IB	5 (50.0)	33 (51.6)	
Lymphovascular invasion			
No	9 (90.0)	51 (79.7)	
Yes	1 (10.0)	10 (15.6)	1.000
No information	0	3 (4.7)	
Tumor grade			
1+2	6 (60.0)	54 (84.4)	
3	4 (40.0)	10 (15.6)	0.087
Tumor size (cm)			
≤5.0	4 (40.0)	37 (57.8)	
>5.0	6 (60.0)	20 (31.3)	0.169
No information	0	7 (10.9)	

[a]Calculated with known values; chi-squared test. BMI: body mass index; HRT: hormone replacement therapy; BCP: birth control pill.

Table S3. differentially expressed genes in relapsed stage I endometrioid endometrial carcinoma (EEC).

Gene symbol	Transcript cluster ID	Fold change (linear)	P-value
PIGR	2453006	80.32	0.022847
KIAA1324	2350489	39.76	0.006474
PGR	3388365	37.72	0.002484
TSPAN8	3461981	12.9	0.008738
GLYATL2	3374517	11.14	0.004712
TFF3	3933536	8.84	0.038142
SPDEF	2951191	7.89	0.002297
NPAS3	3531736	7.04	0.009552
ASRGL1	3333443	6.91	0.015953
BMP7	3910980	-6.22	0.039253
TFPI2	3061621	5.72	0.004644
PTPN13	2734629	-5.66	0.002171
MMP1	3388807	-5.61	0.003296
COM	3461341	5.51	0.009611
FABP4	3142381	-5.42	0.041377
C2orf88	2520069	4.78	0.038539
PORCN	3976639	4.72	0.013822
MLPH	2534252	4.65	0.010532
FREM2	3486096	-4.57	0.032964
ESR1	2931763	4.29	0.030464
FLRT3	3898355	-4.17	0.017044
TPTE2P5	3510963	4.13	0.046755
GJB2	3504193	-4.05	0.007773
KAT7	3725862	-3.87	0.002774
PLAT	3133233	-3.82	0.049117
POC1B-GALNT4	3464967	3.82	0.007329
TRIM29	3394660	-3.82	0.002788
GNG11	3012978	3.71	0.025519
EFEMP1	2554018	-3.68	0.032558
GRIA2	2749222	3.6	0.000893
CGNL1	3595315	3.54	0.008844
GJB6	3504213	-3.42	0.031443
MYCBPAP	3726498	3.38	0.026392
GREB1	2469825	3.35	0.015519
ITGB3	3724545	-3.35	0.046017
MANSC1	3444906	3.35	0.027344
LRIG1	2680591	3.31	0.002196
TESC	3473436	3.26	0.042492
SLC47A1	3713951	3.24	0.013866
PCDHB9	2832403	-3.22	0.011496
SCGB2A1	3333417	3.2	0.036888
SLC40A1	2591837	3.16	0.01553
NDP	4006280	3.11	0.044988
TNS4	3756262	-3.07	0.016052
BMPR1B	2736462	3.05	0.044605
CADPS2	3070309	3.03	0.019059
ANO1	3338293	2.99	0.015161
ITGB6	2583465	-2.98	0.022259
FGFBP1	2761829	-2.95	0.001774
LAMB3	2453793	-2.9	0.006071
TRIB2	2470165	-2.9	0.021404
SLITRK6	3519840	-2.88	0.042736
ELP3	3091628	2.87	0.019614
KRT5	3455186	-2.81	0.012858
PDK4	3062082	2.81	0.030641
HIST1H2BE	2899194	-2.78	0.029645
ITGA3	3726154	-2.78	0.000779
GGTA1P	3223967	2.72	0.029162
NDRG2	3555736	2.69	0.007423
SRD5A3	2727762	2.67	0.041734
SLCO3A1	3608787	-2.66	0.024493
SOX6	3364306	2.64	0.012525
PLK1S1	3879372	2.63	0.046883
CRYL1	3504226	2.61	0.039124
CEP85L	2971564	2.6	0.006997
FAM150B	2537171	2.59	0.034966
ENC1	2862696	-2.58	0.043229
ARF1	2383750	2.55	0.001045
SERINC2	2328273	2.55	0.009207
RHOA	2674367	2.54	0.022288
SFN	2326774	-2.5	0.036577
STX18	2758733	2.48	0.000128
DEPTOR	3113280	2.47	0.037103
FAM174B	3639406	2.41	0.002082
ARF3	3453476	2.39	0.005123
CREB3L4	2359993	2.38	0.030661
HOMER2	3636391	2.37	0.023756
ARHGAP29	2423829	-2.36	0.022853

RPS6KA5	3576284	2.35	0.020296
SHANK2	3380365	2.34	0.022442
CCDC129	2995765	2.33	0.014738
GPR146	2986999	2.33	0.007902
PRPF8	3740634	2.33	0.041549
PSMB2	2406305	-2.33	0.018475
DOCK5	3090512	2.32	0.033191
KLF12	3517793	-2.31	0.036387
MT1L	3662130	2.31	0.042246
GCNT2	2894573	2.3	0.026258
NTPCR	2385696	2.29	0.032421
NRP2	2524301	-2.28	0.015271
HNRNPK	3212360	-2.26	0.015601
KRT17	3757213	-2.25	0.047278
NAAA	2773872	2.25	0.01091
NARS	3809692	-2.25	0.044321
DDAH1	2420832	2.24	0.039771
LCE2B	2359360	-2.24	0.028901
MIPOL1	3532935	2.24	0.000544
NAV1	2374746	-2.24	0.031761
COL17A1	3305081	-2.23	0.038374
PCDHB10	2832423	-2.23	0.014992
STXBP6	3558418	2.23	0.014264
PTAFR	2403446	-2.22	0.043473
HRH1	2610707	-2.21	0.021488
JTB	2436416	2.21	0.040418
TRAF3IP2	2969810	2.21	0.009912
EYA2	3887479	2.2	0.012058
FCGR3B	2440943	-2.2	0.012638
EPDR1	2997907	2.19	0.040568
HSPB6	3859946	2.17	0.034618
KMO	2388085	2.17	0.030701
NRIP1	3925639	2.16	0.038958
OR1E1	3741374	2.16	0.002152
PAM	2822215	2.16	0.017646
SLC4A7	2666904	-2.16	0.005871
USP22	3749652	2.16	0.014121
SLC26A2	2835300	2.15	0.034638
PARK7	2318761	-2.13	0.029836
RGS10	3309602	2.12	0.001416
ZBED5	3362934	-2.12	0.027968
EFNB1	3980170	-2.11	0.044822
C11orf58	3322091	-2.1	0.014968
SH3BGRL2	2914693	2.1	0.020519
C10orf32	3261952	2.09	0.008692
SRPK2	3066297	2.09	0.037238
GLIS3	3197140	2.08	0.038284
HNRNPC	3556268	2.08	0.001615
MARK1	2381177	2.08	0.013714
PAG1	3142217	-2.08	0.012491
POM121	3007438	2.08	0.010436
SLC2A3	3442854	-2.08	0.038021
SLC43A1	3373893	2.08	0.04123
ZNF238	2388794	2.08	0.006731
NUCB2	3322251	2.07	0.041583
OBP2B	3228545	2.06	0.048017
SLC4A4	2730746	2.06	0.008179
TCEB2	3677170	2.06	0.005971
ICA1	3038065	2.05	0.036588
CLOCK	2769947	2.04	0.045442
LOC100287896	3340251	2.04	0.03138
TMEM63A	2458513	2.04	0.020356
EFNA1	2360677	2.03	0.025364
FCGR2A	2363689	-2.03	0.00684
ERGIC1	2841184	2.02	0.042108
KIRREL	2362089	-2.02	0.030272
KRT16	3757177	-2.02	0.022209
STMN3	3914050	-2.02	0.032578
GOLT1A	2451931	2.01	0.018599
ISOC1	2827709	2.01	0.001834
RBM24	2896848	2.01	0.032248

Table S4. Association analyses between *ESR1* gene expression and clinicopathological features of stage I endometrioid endometrial carcinoma (EEC) patients.

Variable	*ESR1* expression median (*GAPDH* units)	P-value[a]
Age		
≤65	0.0054	0.91
>65	0.0036	
BMI		
Adequate	0.0021	0.25
Overweight/obesity	0.0054	
Hypertension		
No	0.0063	0.41
Yes	0.0041	
Diabetes		
No	0.0059	**0.026**
Yes	0.0021	
Pre-menopause		
No	0.0049	0.67
Yes	0.0069	
Pregnancy		
No	0.0101	0.40
Yes	0.0050	
HRT		
No	0.0041	0.65
Yes	0.0031	
BCP		
No	0.0029	0.59
Yes	0.0050	
Stage		
IA	0.0070	0.45
IB	0.0041	
Lymphovascular invasion		
No	0.0054	0.63
Yes	0.0043	
Tumor grade		
1+2	0.0054	0.55
3	0.0030	
Tumor size (cm)		
≤5.0	0.0063	0.99
>5.0	0.0043	

[a]Calculated with known values; chi-squared test. Bold type indicates statistically significant. BMI: body mass index; HRT: hormone replacement therapy; BCP: birth control pill.

Table S5. Correlation analyses between *ESR1* gene expression and the expression of transcription factor genes related to the differentially expressed genes (DEGs) profile in stage I endometrioid endometrial carcinoma (EEC).

Transcriptional factor	Gene symbol	Spearman r	P-value
BACH1	*BACH1*	–0.1758	0.6321
FAC1	*BPTF*	0.1824	0.6138
CEBP	*CEBPB*	–0.01216	0.9803
HNF3	*FOXA1*	0.0304	0.9382
	FOXA2	0.6242	0.0603
	FOXA3	–0.297	0.4069
FREAC2	*FOXF2*	0.2462	0.4918
FOX	*FOXO4*	0.4424	0.2044
HOXA9	*HOXA9*	–0.3333	0.3487
AP1	*JUN*	0.3818	0.2788
LEF1	*LEF1*	–0.3333	0.3487
MAS	*MAS1*	–0.2	0.5837
MEF2	*MEF2A*	–0.103	0.785
	MEF2B	–0.2242	0.5367
	MEF2C	–0.5289	0.1196
	MEF2D	0.01818	0.973
NF1	*NF1*	0.1273	0.733
NFAT	*NFATC1*	–0.1162	0.7493
	NFATC2	–0.2727	0.4483
	NFATC3	–0.2606	0.4697
	NFATC4	–0.2	0.5837
PAX4	*PAX4*	0.4559	0.1861
PITX2	*PITX2*	0.3526	0.3156
AREB6	*PPP1R11*	0.1394	0.7072
SP1	*SP1*	–0.01818	0.973
SREBP1	*SREBF1*	–0.2796	0.4316
TATA	*TBP*	–0.2727	0.4483
E12	*TCF3*	–0.4134	0.2349
TCF4	*TCF4*	0.1273	0.733
p53	*TP53*	–0.3333	0.3487
CHX10	*VSX2*	0.07879	0.8382

Table S6. Impact of *ESR1* expression on the outcome of women with stage I endometrioid endometrial carcinoma (EEC).

Variable	Disease-free survival								Overall survival							
	Univariate				Multivariate				Univariate				Multivariate			
	HR	95%CI		P-value	HR	95%CI		P-value	HR	95%CI		P-value	HR	95%CI		P-value
		Low	High			Low	High			Low	High			Low	High	
Age at diagnosis	0.89	0.38	2.06	0.79					1.07	0.40	2.86	0.88				
Adjuvant treatment	1.09	0.47	2.53	0.82					1.30	0.48	3.50	0.59				
Lymphovascular invasion	1.44	0.48	4.27	0.50					0.89	0.20	3.96	0.88				
Myometrial invasion	1.09	0.47	2.53	0.83					1.30	0.48	3.52	0.59				
Tumor grade	2.46	0.95	6.34	0.06	**3.31**	**1.20**	**9.09**	**0.020**	1.38	0.39	4.86	0.50				
SAH	1.10	0.45	2.71	0.83					1.56	0.50	4.84	0.44				
BMI	3.03	0.70	13.11	0.13	1.24	0.26	5.84	0.78	4.43	0.57	34.0	0.15	2.57	0.32	20.78	0.37
Diabetes	0.14	0.02	1.05	0.05	0.39	0.04	3.20	0.38	0.20	0.02	1.54	0.12	0.50	0.06	4.14	0.52
ESR1 expression	**5.08**	**1.72**	**15.05**	**0.003**	**7.25**	**1.99**	**26.34**	**0.002**	**4.31**	**1.22**	**15.13**	**0.022**	**5.15**	**1.11**	**23.69**	**0.035**

Bold type indicates factors significantly associated with prognosis. HR: hazard ratio; CI: confidence interval; SAH: systemic arterial hypertension; BMI: body mass index.
